# Time for ‘Green’ during COVID-19? Inequities in Green and Blue Space Access, Visitation and Felt Benefits

**DOI:** 10.3390/ijerph18052757

**Published:** 2021-03-09

**Authors:** Thomas Astell-Burt, Xiaoqi Feng

**Affiliations:** 1 Population Wellbeing and Environment Research Lab (PowerLab), Faculty of Arts, Social Sciences and Humanities, School of Health and Society, University of Wollongong, Wollongong, NSW 2522, Australia; xiaoqi.feng@unsw.edu.au; 2 Illawarra Health and Medical Research Institute, University of Wollongong, Wollongong, NSW 2522, Australia; 3 School of Population Medicine and Public Health, Peking Union Medical College, The Chinese Academy of Medical Sciences, Dongcheng District, Beijing 100730, China; 4 National Institute of Environmental Health, Chinese Center for Disease Control and Prevention, Beijing 102206, China; 5 Menzies Centre for Health Policy, University of Sydney, Sydney, NSW 2006, Australia; 6 Faculty of Medicine, School of Population Health, UNSW, Sydney, NSW 2052, Australia

**Keywords:** COVID-19, nature, green space, blue space, accessibility, visitation, preferences, exercise, solace, connection, discovery, lockdown, working from home, remote work, financial difficulty

## Abstract

We hypothesized that visits to green and blue spaces may have enabled respite, connection and exercise during the COVID-19 pandemic, but such benefits might have been inequitably distributed due to differences in financial difficulties, opportunities to work from home, and localized restrictions in spatial mobility generated by ‘lockdowns’. A nationally representative online and telephone survey conducted in 12–26 October on the Social Research Centre’s Life in Australia^TM^ panel (aged ≥ 18 y, 78.8% response, N = 3043) asked about access, visitation, and felt benefits from green and/or blue spaces. Increasing financial difficulty was associated with less time in and fewer visits to green and/or blue spaces, as well as fewer different types visited. Financial difficulty was also associated with feelings that visits to green and/or blue space had less benefit for maintaining social connection. Working from home was associated with more frequent and longer visitation to green and/or blue spaces, as well as discovery of ones previously unvisited. Working from home was also associated with increased levels of exercise and respite resulting from visits to green and/or blue spaces. Residents of Melbourne, a city of 4.9 million who were in ‘lockdown’ at the time of the survey, appeared more likely to benefit from visits to green and/or blue spaces than residents of Sydney, Australia’s largest city at 5.2 million, who were not in lockdown. Residents of Melbourne compared with Sydney reported consistently increased visitation of, discovery of, and greater levels of various felt benefits derived from green and/or blue spaces, including more respite, connection, and exercise. Comparatively shorter distances to preferred green and/or blue spaces and closure of alternative settings at the time of the survey completion in Melbourne compared with Sydney may provide partial explanation, though more acute responses to experiencing green and/or blue spaces within highly cognitively demanding antecedent conditions posed by lockdown are also plausible and warrant further investigation with other health indicators. These results were robust to adjustment for a range of covariates including preferences for natural settings, which were consistently associated with greater levels of green and/or blue space visitation and felt benefits. Collectively, these results indicate that parallel efforts to generate (or renew) felt connection to natural settings, to increase working from home opportunities, and to mitigate financial difficulties may be important to help maximize the population health benefits of urban planning strategies intended to improve the availability of, and to reduce inequities in access to, green and blue spaces. Benefits felt more commonly by people living through lockdown underlines the role previous investments in green and blue space have played in enabling coping during the COVID-19 pandemic.

## 1. Introduction

Unprecedented disruption caused by the COVID-19 pandemic has resulted in millions of people around the world being confined to, and/or working from home [1,2]. Some may have used additional discretionary time not spent commuting to exercise, meet neighbours and seek relief from pandemic-related worries in natural outdoor settings, such as nearby green and blue spaces (e.g., parks, woodlands, beaches and lakes). While it is already known that preferences for natural settings vary and are associated with increased park visitation [3,4], anecdotal [5,6,7] and emerging peer-reviewed evidence [8,9,10,11] indicates many people may also have rediscovered an appetite for nature and its health benefits during this period. For many people, renewal of nature-seeking behaviour may have been motivated by a need for respite, desires to socially connect, and restoration of psychological resources depleted by concerns about the health of others and oneself, diminished job prospects and uncertain financial livelihoods [12,13]. Many others may also have visited green and/or blue spaces as familiar settings in which to psychologically distance themselves from various demanding processes of rapid adaptation, such as near-universal computer-mediated communication for working and home-schooling [14]. 

Though many health benefits of contact with natural settings are increasingly recognised and evidence-based [15,16,17], strict limitations on spatial mobility for long periods in some cities (e.g., Melbourne, Australia [18,19]) may have restricted time in nature to less than might be ideal for generating health benefits [20,21]. Many people may have been dislocated from natural settings beyond permitted mobility catchments they had loved to visit. This is likely to have been felt most severely for socioeconomically disadvantaged groups, given known inequities in access to green and blue spaces [22,23,24,25]. Furthermore, since ability to work from home was skewed towards more affluent groups [26], people on low incomes and/or experiencing financial difficulties who already tend to be in disproportionately poor health [27,28] may have experienced a double disadvantage with comparatively poorer access and less time to visit green and/or blue spaces relative to their more affluent counterparts. 

We hypothesised that (1) those in socioeconomically advantaged positions and/or in (2) occupations permitting working from home during the pandemic may have visited green and/or blue spaces more often and benefitted disproportionately from those visits in comparison with counterparts in socioeconomically less favourable and less flexible and time-poor job circumstances. Furthermore, we also hypothesised (3) people who experienced restricted spatial mobility due to ‘lockdown’ had reduced opportunities to visit and reap benefits from green and/or blue space. In this study, we provide a first look at the situation in Australia using a nationally representative survey conducted in October 2020. Australia’s relatively successful response to the COVID-19 pandemic in comparison to other English-speaking high income countries has included a second-wave of infections in Victoria [29] and a near-three month lockdown of the city of Melbourne [18,19], providing grounds for a natural experiment that could provide insights into how the pandemic has influenced access, visitation, and felt benefits from green and blue spaces.

## 2. Method

### 2.1. Data

This study involved survey of Social Research Centre’s Life in Australia^TM^ panel between 12 and 26 October 2020. Members of this panel, aged ≥ 18 y, had originally been randomly recruited in 2016 via their landline or mobile phone using a dual-frame random digit dialling design (RDD) with a 30:70 split between landline and mobile phone sample frames. An alternating next or last birthday approach was used to select respondents via the landline method from households where there were at least two residents in scope. The phone answerer was the selected respondent via the mobile phone method. In each case, invitation to join the panel was for one member per household only. The panel was refreshed in 2018 using mobile phone RDD only and again in 2019 with online-only participants using a G-NAF (Geocoded National Address File) sample frame and push-to-web methodology. In each of these cases, refreshment was required to balance demographics of the panel with respect to the Australian population. As of October 2020 the panel included N = 3860 active members located across the following geographical areas: (1) Greater Sydney; (2) Rest of New South Wales (NSW); (3) Greater Melbourne; (4) Rest of Victoria (Vic.); (5) Greater Brisbane; (6) Rest of Queensland (Qld.); (7) Greater Adelaide; (8) Rest of South Australia (SA); (9) Greater Perth; (10) Rest of Western Australia (WA); (11) Greater Hobart; (12) Rest of Tasmania (Tas.); (13) Greater Darwin; (14) Rest of Northern Territory (NT); (15) Australian Capital Territory (ACT).

The Social Research Centre’s contact methodology for online panel members involved an initial survey invitation via email and SMS (where available). This was followed by several reminders via email and a reminder SMS. Telephone non-response of panel members took place in week 2 of the survey period and involved reminder calls encouraging completion of the online survey. SMS was also used where available for contacting offline panel members, followed by an extended call-cycle and reminder SMS over the two-week survey period. Messages were left on answering machines and voicemails. Participation was in the English language only. All interviewers and supervisors selected by the Social Research Centre to conduct the survey had received training in the Life in Australia^TM^ panel, survey procedures and sample management protocols, respondent liaison procedures, strategies to maintain co-operation, and detailed examination of the survey questionnaire developed by the researchers. 

Ethical approval for the survey was granted by the University of Wollongong HREC. Approximately 78.8% (N = 3043) of the Life in Australia^TM^ panel participated in our survey (95.0% completing online, 5.0% completing via telephone). Completion rates were 78.8% overall, 80.0% for online panel members, and 62.3% for offline panel members. An incentive of a supermarket or department store gift card, direct payment into a PayPal account, or donation to a designated charity was offered to all panel members to the value of AUD $10.00 each. The survey took 15.2 min on average to complete, with mean completion times of 14.9 min online and 22.0 min by telephone. 19.9% of panel members could not be contacted during the survey period and 1.3% of invited members decline participation. Response propensity weights were developed using logistic regression by the Social Research Centre to limit the impact of non-participation on sample representativeness. These weights took into account location, age group, gender, annual household income, citizenship status, language(s) spoken other than English, country of birth, Aboriginal or Torres Strait Islander status, number of adults and children in the household, employment status, marital status, highest education, television viewing and internet browsing habits, smoking and drinking status, general health, life satisfaction, early adopter status, caregiving, disability status, volunteer status, concession card status, and telephony status. 

### 2.2. Green Space Access, Visitation and Felt Benefits

A range of questions were asked of participants about their access to green and blue space. Access focussed on walking with the question “*How long [in minutes] would it take for you to walk to the nearest green space and/or blue space from your home?*” Participants were then asked “*Is the green space and/or blue space nearest your home the one you prefer to visit locally and most often? [yes or no]*” Those participants responding negatively were then asked “*How long [in minutes] would it take for you to walk to your preferred local green space and/or blue space from your home?*” A variable was derived to record the difference in minutes of walking from home to the nearest green and/or blue space and to that which participants preferred. These questions specified walking as the mode of transport to enable comparisons, even if participants typically selected to travel to natural settings by other means. The typical travel mode to visit green and/or blue space was also asked. 

A second set of questions asked participants about their contact with green and/or blue spaces. Visit frequency was asked using the following question: “*In the past four weeks (including the weekends), how often have you visited your preferred local green space and/or blue space? [Almost daily, 1–4 times weekly, 2–3 times in the past month, once or less in the past month, never]*” To gauge cumulative time spent in natural settings across a week the participants were asked “*Approximately how many hours did you spend in green spaces and/or blue spaces in total over the last 7 days?*” They were also requested to select different types of natural settings where these visits took place, including playing fields, ovals or bowling greens, small parks, large parks, nature reserves, woodlands or forests, botanic gardens, farmland, mountains, hill or moorland, greenery by a river, lake or canal, a beach, or other. If people reported no visits to preferred green and/or blue spaces in the four weeks prior to the survey date, potential reasons were explored with the question “*What are the reasons you have not visited your preferred local green space and/or blue space in the last four weeks?*”

Finally, participants were asked to reflect on how their use of, and felt benefit from, visiting green and blue space may have changed since the COVID-19 pandemic began in five questions. Answered with a Likert scale (strongly disagree, disagree, neither agree nor disagree, agree, strongly agree), these questions were as follows: “*Since the COVID-19 pandemic and social distancing began in Australia, to what extent, if at all, do you agree or disagree with each of the following statements? (A) I now visit green spaces and/or blue spaces more often than before the COVID-19 pandemic. (B) Green spaces and/or blue spaces have helped me to stay connected with my neighbours during the COVID-19 pandemic. (C) Green spaces and/or blue spaces have brought me solace and respite in these challenging times. (D) I now walk and/or exercise in green spaces and/or blue spaces more frequently than before the COVID-19 pandemic. (E) I have discovered and visited green spaces and/or blue spaces that I hadn’t had the opportunity to visit before the COVID-19 pandemic*”.

### 2.3. Covariates

Patterns of green space access, visitation and felt benefits were examined with respect to a range of personal, socioeconomic, and household-level covariates measured in the survey. Personal covariates include gender, age, country of birth (coded as Australia, overseas predominantly non-English speaking, and overseas predominantly English-speaking), and the 6-item ‘Nature-Relatedness Scale’ (NRS) [30]. A measure of NRS was used to take into account variations in preferences for spending discretionary time in natural settings within the population and thereby to differentiate between increased levels of nature-seeking behaviour that may be attributable to this underlying intrinsic motivation from other enabling and constraining factors. 

Enabling and constraining factors assessed included socioeconomic variables including highest educational qualification, annual household income (AUD $ before tax), perceived financial situation *(“How well would you say you yourself are managing financially these days? Would you say you are… living comfortably, doing alright, just about getting by, finding it quite difficult, finding it very difficult?”)* and economic status (employed, unemployed, retired, long-term sick or disabled, other). Given substantial shifts to working from home in Australia during the COVID-19 pandemic, the ‘employed’ category was segmented after survey completion using responses to the following question: “*During the last four weeks how often did you work at home? [Always, often, sometimes, never]*” Household variables included whether a person was living on their own, with another adult and/or with children, the type of household (house, flat, farmhouse, retirement community, other), whether or not a dog was present in the household, and if the household had access to an outdoor space (private garden, communal garden, or other, e.g., balcony).

### 2.4. Statistical Analysis

Analysis focussed on a complete-case sample of N = 2697. Frequency tables, percentages and means were calculated to describe each of the green space access and use variables with respect to all covariates. These descriptive statistics were conducted in Stata v.14 (StataCorp, College Station, TX, USA) using ‘SVY’ to incorporate probabilistic weights to provide nationally representative results. Multilevel logistic and Poisson regressions were then fitted in MLwIN v3.02 (Centre for Multilevel Modelling, University of Bristol) [31] estimated with Markov Chain Monte Carlo (MCMC) [32] method with 5000 iterations burn-in and 50,000 chain-length. Weights were not applied in these models as they were not supported for MCMC-estimated models fitted in MLwIN. Participants were set at level 1, nested within a high-level geographical classification at level 2 that distinguished major cities from other areas of states and territories. Further analysis involved models contrasting participants residing in the five most populous cities in Australia (Sydney, Melbourne, Brisbane, Adelaide, and Perth), with cities fitted as a fixed effect variable. Sydney was set as the reference group to permit contrasts with Melbourne in particular, whose residents was in lockdown during the survey.

## 3. Results

### 3.1. Access to Green and/or Blue Space

Table 1 shows participants walked about 8.3 min on average (95% CI 7.8, 8.7) to the nearest green and/or blue space. Mean walking times were higher before and after adjustment for males (vs. females), the retired or long-term sick or disabled (vs. employed, not working from home), those perceiving their financial situations as less than comfortable, or in the high nature relatedness score category. Minutes walked to the nearest green and/or blue space also tended to increase with age. Participants with higher educational qualifications or higher incomes tended to walk substantively fewer minutes to the nearest green and/or blue space. Approximately 53.9% (95% CI 51.5, 56.4) stated a preference for the green or blue space nearest their home. Before and after adjustment, preference for the nearest green or blue space was lower among males and participants with higher educational qualifications. Preference for the nearest green or blue space was higher among those over 35 years old, and/or with higher nature relatedness scores. Preference for the nearest green/blue space was not associated with economic status, perceived financial situation, or income. Among the subset of participants who did not prefer to visit the nearest green/blue space (N = 1122), it took an additional 22.0 min (95% CI 20.2, 23.9) to walk to the one they preferred to visit locally and most often (Table 2). Before and after adjustment, minutes of additional walking were higher among males, the unemployed, retirees, long-term sick or disabled, those with a Bachelor degree, annual household income between AUD 51,000 and AUD 100,000, or those whose perceived financial situation was less than comfortable. Retirees appeared to have lower mean minutes walked before adjustment, but this pattern inverted after adjustment. Mode of travel to visit preferred natural settings was on foot (56.0%), followed by private car (37.7%) and cycling (3.3%). Less than 1% travelled by bus or train.

### 3.2. Green and/or Blue Space Visitation

Table 3 shows 3.2 was the mean number of hours spent over the last 7 days in green and/or blue space (95% CI 3.1–3.3). Before and after adjustment the mean number of hours was lower among males, the long-term sick or disabled, or in less than comfortable perceived financial situations. Mean hours in a green or blue space tended to be higher with age, employed persons working constantly from home (vs. employed not working from home), or with higher nature relatedness scores. 

The mean number of different types of nature visited in the last 7 days was 1.6 (95% CI 1.5–1.7). The most popular options were: small parks (40.1%); playing fields, ovals and bowling greens (24.7%); large parks (24.0%); nature reserves, woodlands and forests (20.7%); greenery by a river, lake or canal (18.2%); and beaches (13.0%).

Before and after adjustment, the mean number of different types of green and/or blue space visited was lower among the long-term sick or disabled, and those who felt their financial situation difficult. The mean number of different types of natural setting did not vary with age, but was higher among those employed and working from home often or always, with bachelor degree or higher, incomes above AUD 150,000, and with higher nature relatedness scores.

Approximately 52.0% (95% CI 49.6, 54.4) of the sample visited their preferred green or blue space at least once a week in the last 4 weeks (Table 4). The odds of visiting at least once a week were higher among participants aged 55–64 y, the unemployed, retirees, those with a bachelor’s degree or higher, and/or with higher nature-relatedness scores. Odds of visiting preferred nearby natural settings were lower for the long-term sick or disabled, and those who felt their financial situations were less than comfortable.

### 3.3. Changes in Use and Felt Benefits from Visiting Green or Blue Spaces

Table 4 also shows the percentage of the sample claiming to visit green and blue spaces more often than they did prior to the COVID-19 pandemic was 27.6% (25.5, 29.9). This was slightly higher among males than females and in older age groups. Higher odds were observed for people employed and working from home often or always, and participants with higher nature-relatedness scores. Increased levels of visitation did not appear to be associated with measures of socioeconomic circumstances.

About 25.7% of the sample felt they had been able to reconnect with neighbours thanks to visiting nature during the COVID-19 pandemic (Table 5). Levels of felt reconnection were higher among males and older adults, with a Bachelor’s degree or higher, and with higher nature-relatedness scores. Participants who perceived their financial situation as difficult had lower odds of felt social connection resulting from visits to green or blue spaces.

Feelings of solace and respite from visiting green or blue spaces during the COVID-19 pandemic were reported by 53.7% (95% CI 51.3, 56.1). The odds of feeling solace and respite were higher among males, participants working from home, retirees, those with a Bachelor’s degree or higher, and especially with higher nature-relatedness scores. The odds were of feeling solace and respite lower with age, especially among the ≥75 s.

Table 6 shows 28.2% of the sample reported walking and/or exercising more often in green and/or blue spaces since before the COVID-19 pandemic (95% CI 26.0, 30.5). Odds of walking or exercising more often in green or blue space were lower with age, though also higher among people who were working from home often or always, and those with higher nature-relatedness scores.

Discovery and use of green or blue spaces that participants had not had the opportunity to visit prior to the COVID-19 pandemic was 27.1% of the sample (24.9, 29.5). Higher odds were reported among males, those consistently working from home, and participants with higher nature-relatedness scores. Lower odds were reported with age.

### 3.4. Geographic Variation

**Figure 1:**
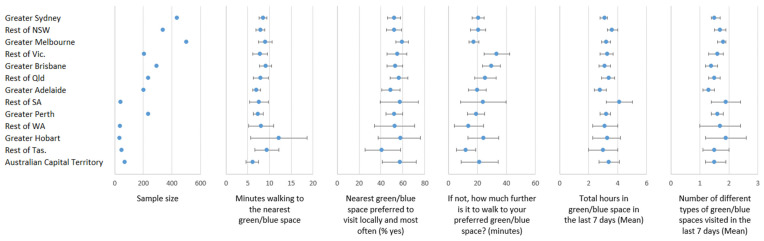
Geographical differences in access to and visitation of the nearest and preferred green or blue spaces during the COVID-19 pandemic (weighted for representativeness, unadjusted for covariates).

**Figure 2:**
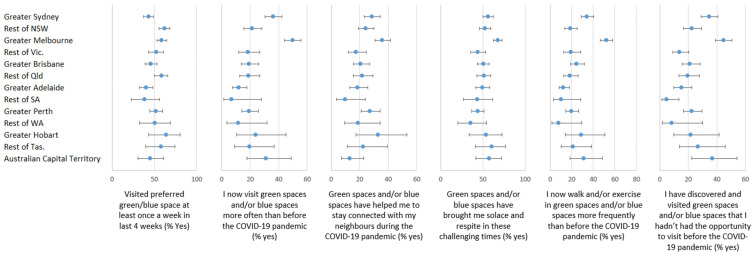
Geographical differences in access to and visitation, changes in visits and felt benefits of preferred green or blue spaces during the COVID-19 pandemic (weighted for representativeness, unadjusted for covariates).

Figure 1 reports over half the sample (54.8%) were resident in Greater Sydney (16.1%), the rest of New South Wales (NSW, 12.5%), Greater Melbourne (18.6%), or in the rest of Victoria (Vic, 7.6%). Greater Darwin and the rest of the Northern Territory are not reported as their numbers were low, representing 0.55% of the sample as a whole. The descriptive values presented in Figure 1 and Figure 2 are weighted for representativeness (see Methods), but not adjusted for covariates. Between-area variances estimated from variance component models are reported in Table 1, Table 2, Table 3, Table 4, Table 5 and Table 6, along with percentage change in variances observed after adjustment for covariates. Geographical variation in mean minutes to the nearest green and/or blue space was low across most states and territories, with slightly more substantial deviations for areas with small sample sizes only, resulting in wider 95% confidence intervals (e.g., Greater Hobart). There was similarly sparse evidence of geographic variation in preferences for the nearest green and/or blue space, or the additional minutes walked to the preferred green or blue space if not the nearest one. About 61.4% of geographic variation in cumulative hours spent visiting green and/or blue space in the last 7 days was explained by individual differences. Little geographic variation in the variety of green/blue spaces was evident at this spatial scale. 

Figure 2 shows the percentage of participants visiting their preferred green and/or blue space at least once a week in the last 4 weeks was at 43.1% among residents of Sydney compared with 62.0% in the rest of NSW, and 58.8% in Melbourne despite the limitations on spatial mobility. 58.3% in the rest of Queensland but down to 46.0% in Brisbane, 40.1% in Adelaide and 38.4% in the rest of South Australia (SA). Adjustment for individual differences did not explain these between-area differences. Geographic variation was most evident for the reporting of increased green and/or blue space visitation, increased physical activity and increased levels of discovery of new nature since the beginning of the COVID-19 pandemic in Australia. Increased levels of green and/or blue space visitation was notably higher in Melbourne (49.7%) compared with Sydney (36.1%), Adelaide (11.9%) and Perth (19.3%) and each were somewhat higher relative to the remainder of the states in which they were situated. Individual differences explained approximately 30.8% of this geographic variation in increased visitation levels. 

Despite being in lockdown, residents of Melbourne reported the highest levels of felt connection with neighbours (35.9%), solace and respite (67.8%), increased levels of walking and other exercise in green and/or blue spaces (52.1%), and discovery of new natural settings (44.6%), compared to peers in most other areas of Australia. Adjusting for individual factors explained about 30% of the geographic variation in increased levels of walking and exercise, and about 61.4% of geographic variation in the discovery of new green and/or blue spaces.

### 3.5. Between-City Differences

**Figure 3:**
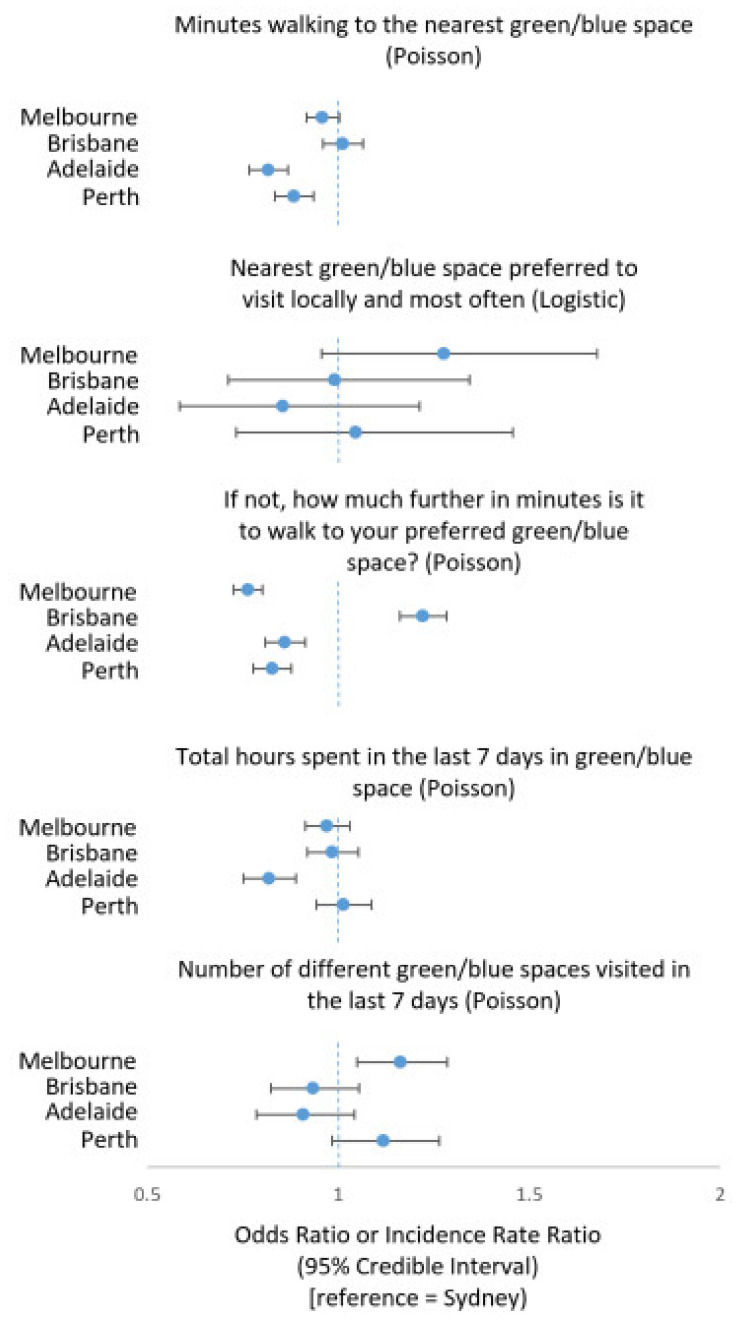
Differences between Australia’s most populous cities in access to and visitation of the nearest and preferred green or blue spaces during the COVID-19 pandemic (multilevel Poisson and logistic regressions).

Adjusted analysis of the five most populous cities in Australia (Figure 3) indicated fewer minutes spent walking to the nearest green and/or blue space for people in Adelaide (IRR = 0.82, 95% CI = 0.77, 0.87) and Perth (IRR = 0.88, 95% CI = 0.83, 0.94) compared to Sydney. Odds of preferring the nearest green or blue space were higher in Melbourne compared to Sydney (OR = 1.28, 95% CI = 0.96, 1.68), albeit with some imprecision. Among participants not preferring their nearest green or blue space, those in Melbourne (IRR = 0.76, 95% CI = 0.73, 0.80), Adelaide (IRR = 0.85, 95% CI = 0.81, 0.91) and Perth (IRR = 0.83, 95% CI = 0.78, 0.88) had fewer minutes further to walk to their nearest one compared to people in Sydney, whereas those in Brisbane tended to walk further (IRR = 1.22, 95% CI = 1.16, 1.28).

Adjusted analysis of the five most populous cities indicated fewer hours spent in green and/or blue space in the last 7 days among people in Adelaide compared to Sydney (IRR = 0.82, 95% CI = 0.75, 0.89), but no major differences with other cities. People in Melbourne tended to visit a greater variety of natural settings than their counterparts in Sydney (IRR = 1.16, 95% CI = 1.05, 1.29). Odds of visiting preferred green and/or blue space at least once a week for the past 4 weeks were notably higher in Melbourne (OR = 2.10, 95% CI = 1.56, 2.76) and Perth (OR = 1.50, 95% CI = 1.05, 2.09) compared with Sydney.

**Figure 4:**
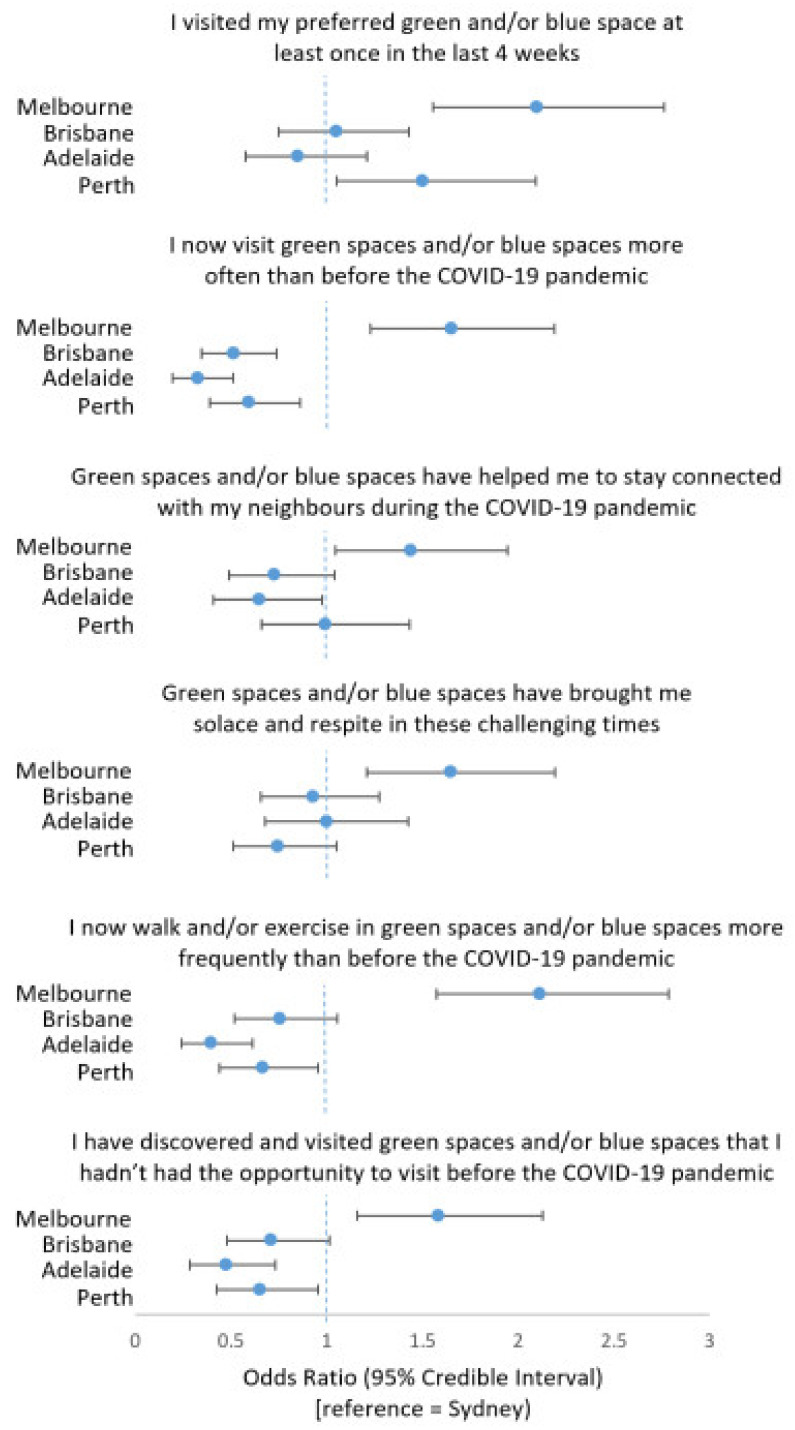
Differences between Australia’s most populous cities in visitation, changes in visits and felt benefits of preferred green or blue spaces during the COVID-19 pandemic (multilevel logistic regressions).

Adjusted between-city analysis with Sydney as the reference group in Figure 4 indicated participants in Melbourne had higher odds of visiting green and/or blue spaces more often (OR = 1.66, 95% CI = 1.23, 2.19), felt those visits kept them socially connected (OR = 1.44, 95% CI = 1.05, 1.95), experienced solace and respite from those visits (OR = 1.65, 95% CI = 1.21, 2.19), reported walking and exercising more frequently in green and/or blue spaces than before the pandemic (OR = 2.11, 95% CI = 1.58, 2.79), and discovered new natural settings they had not previously visited (OR = 1.59, 95% CI = 1.16, 2.13).

Compared to Sydney, people in Brisbane (OR = 0.52, 95% CI = 0.34, 0.73), Adelaide (OR = 0.33, 95% CI = 0.19, 0.51) and Perth (OR = 0.60, 95% CI = 0.39, 0.86) had lower odds of visiting natural settings more often since the pandemic began. People in Adelaide reported lower odds that visits to green and/or blue spaces had helped to keep them connected (OR = 0.65, 95% CI = 0.41, 0.98) and lower odds of walking or exercising in them more often (OR = 0.40, 95% CI = 0.24, 0.61), as well as lower odds of discovering new natural settings (OR = 0.48, 95% CI = 0.28, 0.73) compared with Sydney-based participants. Participants in Perth also had lower odds of reporting increases in exercising in green and/or blue spaces since before the pandemic (OR = 0.67, 95% CI = 0.44, 0.95) and discovering new natural settings (OR = 0.65, 95% CI = 0.43, 0.96) compared to people in Sydney.

## 4. Discussion

Various findings from our study provide new evidence and indicative avenues for further research on inequities in green and blue space contact and consequences for population health and health inequity during the COVID-19 pandemic and beyond. Evidence was found in support of our first hypothesis with respect to perceived financial wellbeing and education level. People in socioeconomically disadvantaged circumstances tended to visit green and/or blue spaces less often, and feel less benefit from those visits, in comparison to their more affluent counterparts. For example, people reporting financial difficulty spent less time overall and visited fewer different types of green and blue spaces, visited their preferred natural settings less often, and were less likely to feel those visits kept them socially connected compared with financially comfortable counterparts. Moreover, people with university degrees compared to those with less than a year 12 education tended to report better access to, and were more likely to derive respite and social connection from visiting green and/or blue spaces. To what extent these socioeconomic inequities in felt benefits of green and/or blue space are attributable to known socioeconomic differences in the perceived quality of those natural settings [33,34], and/or are a consequence of disproportionately greater suffering experienced by people in disadvantaged circumstances before and during the COVID-19 pandemic requires further investigation.

Evidence was consistent for our second hypothesis: people who were able to work from home reported more frequent levels of visitation, longer cumulative visit times, visits to a greater variety of settings, discovery of green and/or blue spaces they had not previously encountered, and felt those visits enabled more frequent exercise and supported feelings of solace and respite, in comparison to those unable to work from home and independent of confounding factors such as income and education. This despite no differences in access to green and/or blue spaces between those working from home and those who had not been permitted to do so. There was no clear association between working from home and feelings that contact with natural settings had supported maintenance of social connection with neighbours. This may be, to a potentially large extent, a consequence of social distancing behaviours when outdoors. Survey-wording may also play a role, since for many people, contact with nature may not have helped them “*to stay connected with… neighbours*” if those people did not know their neighbours before the COVID-19 pandemic. This does not dismiss the possibility of *new* relationships developing. Accordingly, these results indicate the need to examine associations between green space, social capital and related societal issues (e.g., loneliness [35]) in future work set during and after the COVID-19 pandemic.

In contrast to our first and second hypothesis, no evidence was found to indicate that Melbourne residents—who were in an extended ‘lockdown’ during the survey—were disadvantaged in terms of access, visitation and felt benefits from green and/or blue space relative to counterparts living in Australia’s four other most populous cities. In fact, Melbourne residents compared to those in Sydney consistently reported increased visits and discovery of new green and blue spaces, and those visits facilitated increased levels of walking and exercise, social connection and respite during the pandemic. From this, we hypothesise that the antecedent condition of the ‘lockdown’ may have generated circumstances in Melbourne wherein experience of visiting green and/or blue spaces became especially acute and meaningful. Such circumstances were likely entwined with closure of other settings where people may usually derive some form of exercise, connection and relief, such as gyms. Another contributing factor is likely to have been that Melbourne residents, compared to those in Sydney, lived closer and were notably more likely to have visited their preferred natural settings at least once a week in the past month, visited a wider range of green and/or blue spaces, and spent similar amounts of time in nature in the past week. Maintenance of visitation despite lockdown is likely to have been facilitated by relatively shorter distances to the nearest green and/or blue space in Melbourne compared with Sydney. Moreover, evidence indicated that Melbourne residents were not only more likely to prefer the green and/or blue spaces nearest their home, but also had shorter distances to walk if they preferred another further afield. Previous work indicates generally more equal distributions of green space across socioeconomic indicators in Melbourne [22,36]. In short, contact with natural settings seems to have been maintained, and perhaps even intensified in its felt potency, for Melbourne residents during lockdown relative to their peers in Sydney who had no comparable spatial mobility restrictions. The potential impacts of this ‘natural experiment’ on associations between green and/or blue space and other health and behavioural outcomes warrants examination.

One more result merits special attention. Participants with stronger preferences towards natural settings consistently reported greater levels of visitation and felt benefit relative to their peers with lower so-called ‘nature relatedness’ scores. Supply side interventions such as urban greening strategies and improvements in access to blue spaces are no ‘one-size-fits-all’ panacea. Previous literature indicates people with more positive attitudes towards natural settings tend to visit parks more frequently [3,4]. Our results indicate that many people, for whatever reason they might be less attracted by natural settings, may not have derived benefits from nearby green and/or blue spaces during the pandemic comparable to those with more positive attitudes. This might reflect negative attitudes towards and avoidance of natural settings in general, possibly related to unfavourable prior experiences [37] and/or dismay at the condition of those spaces [38]. In some cases perhaps it may also reflect an absence of the types of preferential green and/or blue spaces that not only permit restorative processes to occur, but actively promote feelings of awe, wonder, and shared moments of intimacy whether that be with other humans, resident wildlife, or in solitude [39]. More research designed to enhance understandings of the heterogeneity in appreciation, or lack thereof, for nearby natural settings during the pandemic and in the years ahead merits investment if potential supply and demand-side interventions, such as ‘nature-prescriptions’ [40], are to be consequential beyond those already with an appetite for the ‘great outdoors’.

Strengths of the study include a high response rate and a sample with coverage across all states and territories that enabled nationally representative estimates. Coverage of the five most populous cities was also a strength, especially as it permitted comparison of Melbourne, in ‘lockdown’ during the survey, with other cities like Sydney, which was not. The study is limited by cross-sectional design and self-reported data. Some previous research indicates a mismatch between perceived and actual access to green space [41]. However, this contrast tends to ignore personal preferences that may be crucial to whether a particular green and/or blue space is visited. Thus, the range of questions asked on this issue was a strength, enabling differentiation between the nearest green and/or blue spaces from those which participants actually preferred to visit locally and most often.

## 5. Conclusions

In conclusion, people with greater socioeconomic disadvantage, such as those experiencing financial difficulty, were less likely to visit green and blue spaces during the COVID-19 pandemic. Even when they did, they were less likely to derive benefits from those visits. People who were able to work from home did accrue benefits from contact with natural settings, especially in terms of respite and exercise, though the lack of reported increase in social connection merits further inquiry. Lastly, rather than being disadvantaged by the experience of ‘lockdown’ with respect to visiting natural settings, residents of Melbourne tended to visit them more frequently and reap greater benefits from green and blue spaces compared with their peers in Sydney. This provides foundation for further research to explore potential felt benefits of green and/or blue spaces for other aspects of health and wellbeing during the COVID-19 pandemic within the context of this ‘natural experiment’. Overall, these findings underline the value of implementing strategies that equalise access to green and blue spaces in so that everyone has opportunities to benefit, while also highlighting that parallel efforts to generate (or renew) felt connection to natural settings, to increase working from home opportunities, and to mitigate financial difficulties may also be important to help maximize the population health benefits for all.

## Figures and Tables

**Table 1:** Associations with access to the nearest and preferred green or blue spaces during the COVID-19 pandemic.

	N	%	Minutes Walking to the Nearest Green/Blue Space		Nearest Green/Blue Space Preferred to Visit Locally and Most Often	
			Mean (95% CI)	IRR (95% CrI)	% Yes (95% CI)	OR (95% CrI)
Full sample	2697	100	8.3 (7.8, 8.7)		53.9 (51.5, 56.4)	
***Fixed Effects*** **Gender (ref: Female)**	1217	45.1	7.5 (7.0, 8.0)		56.6 (53.1, 60.0)	
Male	1483	54.9	9.0 (8.2, 9.8)	1.20 (1.16, 1.23)	51.3 (47.9, 54.7)	0.84 (0.71, 0.99)
**Age group (ref: 18–24)**	103	3.8	8.5 (6.1, 10.9)		47.4 (37.0, 57.9)	
25–34	356	13.2	7.9 (6.8, 9.0)	1.04 (0.96, 1.13)	50.8 (44.5, 57.0)	0.95 (0.58, 1.50)
35–44	448	16.6	8.8 (7.6, 9.9)	1.14 (1.05, 1.24)	52.1 (46.3, 57.8)	1.23 (0.75, 1.92)
45–54	458	17.0	8.0 (7.0, 8.9)	1.13 (1.04, 1.23)	51.1 (45.7, 56.4)	1.13 (0.69, 1.77)
55–64	549	20.3	7.8 (7.1, 8.4)	1.19 (1.09, 1.29)	54.4 (49.5, 59.3)	1.19 (0.72, 1.85)
65–74	541	20.0	8.3 (7.5, 9.2)	1.18 (1.08, 1.28)	61.2 (56.1, 66.0)	1.28 (0.73, 2.12)
75+	245	9.1	9.1 (7.8, 10.5)	1.34 (1.21, 1.48)	70.9 (63.5, 77.3)	2.13 (1.11, 3.76)
**Economic status (ref: Employed, not working from home)**	680	25.2	7.9 (7.1, 8.7)		51.7 (47.0, 56.4)	
Employed, working from home sometimes	285	10.6	7.2 (6.1, 8.3)	0.89 (0.84, 0.94)	44.9 (37.9, 52.0)	0.84 (0.62, 1.12)
Employed, working from home often	201	7.4	8.1 (6.3, 9.9)	0.99 (0.93, 1.04)	53.7 (45.0, 62.1)	1.13 (0.80, 1.56)
Employed, working from home always	380	14.1	7.9 (6.9, 8.9)	1.02 (0.97, 1.07)	53.7 (47.2, 60.0)	0.91 (0.69, 1.19)
Unemployed	188	7.0	9.9 (8.0, 11.7)	1.06 (1.01, 1.12)	56.9 (47.9, 65.4)	1.09 (0.75, 1.53)
Retired	787	29.2	8.6 (7.9, 9.3)	1.00 (0.95, 1.05)	63.2 (59.0, 67.1)	1.16 (0.83, 1.59)
Long-term sick or disabled	73	2.7	8.9 (6.7, 11.0)	1.12 (1.04, 1.21)	46.6 (32.6, 61.1)	0.75 (0.43, 1.21)
**Highest qualification (ref: <Year 12)**	345	12.8	9.5 (8.3, 10.6)		62.9 (56.9, 68.5)	
Year 12	332	12.3	8.4 (7.0, 9.7)	0.87 (0.83, 0.92)	50.4 (44.0, 56.8)	0.65 (0.46, 0.89)
Certificate or advanced diploma	744	27.6	8.3 (7.5, 9.1)	0.95 (0.91, 0.99)	55.6 (51.4, 59.7)	0.82 (0.61, 1.08)
Bachelor degree or graduate certificate	859	31.8	7.2 (6.5, 7.8)	0.85 (0.81, 0.89)	48.6 (43.9, 53.3)	0.59 (0.44, 0.77)
Postgraduate qualification	420	15.6	8.3 (7.1, 9.5)	0.90 (0.86, 0.95)	52.7 (46.6, 58.8)	0.66 (0.47, 0.90)
**Annual household income before tax (AUD; ref: <AUD 50,000)**	776	28.7	9.6 (8.6, 10.6)		60.2 (55.6, 64.6)	
AUD 51,000–AUD 100,000	842	31.2	9.1 (8.1, 10.1)	0.98 (0.94, 1.02)	52.6 (48.2, 56.9)	0.87 (0.69, 1.09)
AUD 101,000–AUD 150,000	468	17.3	7.2 (6.4, 7.9)	0.90 (0.86, 0.95)	54.6 (48.8, 60.2)	0.99 (0.73, 1.31)
>AUD 150,000	441	16.3	6.2 (5.5, 7.0)	0.84 (0.80, 0.89)	47.1 (41.2, 53.1)	0.85 (0.60, 1.15)
**Financial situation (ref: Comfortable)**	825	30.6	6.7 (6.1, 7.2)		53.6 (49.2, 58.0)	
Doing ok	1156	42.8	8.5 (7.7, 9.2)	1.16 (1.12, 1.20)	53.6 (49.9, 57.4)	1.00 (0.82, 1.22)
Getting by	491	18.2	9.5 (8.5, 10.6)	1.27 (1.22, 1.33)	55.6 (49.9, 61.1)	1.03 (0.79, 1.33)
Difficult or very difficult	228	8.4	9.5 (7.8, 11.2)	1.24 (1.17, 1.31)	52.9 (44.9, 60.8)	0.92 (0.65, 1.28)
**Nature relatedness scale (ref: Tertile 1 (1.0–3.4))**	884	32.7	7.8 (7.3, 8.4)		49.4 (45.7, 53.1)	
Tertile 2 (3.5–4.0)	1082	40.1	8.0 (7.0, 8.9)	0.94 (0.91, 0.97)	56.3 (51.8, 60.7)	1.23 (1.01, 1.48)
Tertile 3 (4.1–5.0)	734	27.2	9.4 (8.3, 10.5)	1.06 (1.03, 1.10)	59.3 (54.7, 63.8)	1.30 (1.05, 1.57)
***Random Effects*** Unadjusted mean (95% CrI) ^†^			0.05 (0.02, 0.12)		<0.01 (<0.01, 0.03)	
Adjusted mean (95% CrI)				0.03 (0.01, 0.09)		<0.01 (<0.01, 0.04)
Percentage reduction in variance				27.5		-

IRR: Incidence Rate Ratio. OR: Odds Ratio. 95% CrI: 95% Credible Interval. Probabilistic weights applied to descriptive means, percentages and 95% CI (95% Confidence Intervals). ^†^ estimates derived from Variance Components Model. Models additionally adjusted for country of birth, household structure, household type, access to a private garden or balcony, and dog ownership.

**Table 2:** Associations with additional walking minutes to the preferred green or blue spaces, if the one nearest is not preferred.

	How Much Further Is It to Walk to Your Preferred Green/Blue Space, If Not the Nearest? (Minutes)			
	N	%	Mean (95% CI)	IRR (95% CrI)
Full sample	1122		22.0 (20.2, 23.9)	
***Fixed Effects*** **Gender (ref: Female)**	484	43.1	20.6 (17.9, 23.2)	
Male	638	56.9	23.3 (20.7, 25.9)	1.05 (1.02, 1.08)
**Age group (ref: 18–24)**	41	3.7	23.7 (15.1, 32.4)	
25–34	173	15.4	25.0 (20.2, 29.7)	1.01 (0.94, 1.09)
35–44	195	17.4	25.6 (21.7, 29.6)	0.96 (0.89, 1.03)
45–54	205	18.3	23.9 (19.7, 28.0)	0.85 (0.79, 0.92)
55–64	233	20.8	17.2 (14.2, 20.3)	0.71 (0.66, 0.76)
65–74	207	18.5	16.2 (12.6, 19.8)	0.57 (0.52, 0.62)
75+	68	6.1	12.2 (7.6, 16.8)	0.41 (0.37, 0.46)
**Economic status (ref: Employed, not working from home)**	294	26.2	22.9 (19.0, 26.7)	
Employed, working from home sometimes	140	12.5	20.9 (16.2, 25.6)	0.98 (0.94, 1.03)
Employed, working from home often	89	7.9	21.5 (15.5, 27.5)	1.01 (0.96, 1.07)
Employed, working from home always	173	15.4	17.3 (14.1, 20.5)	0.81 (0.77, 0.85)
Unemployed	75	6.7	27.1 (20.0, 34.3)	1.08 (1.02, 1.14)
Retired	280	25.0	17.0 (13.9, 20.2)	1.35 (1.27, 1.42)
Long-term sick or disabled	33	2.9	38.6 (24.8, 52.5)	1.46 (1.36, 1.57)
**Highest qualification (ref: <Year 12)**	112	10.0	21.0 (15.4, 26.6)	
Year 12	138	12.3	21.3 (17.1, 25.5)	0.97 (0.92, 1.03)
Certificate or advanced diploma	283	25.2	22.8 (19.4, 26.2)	1.02 (0.98, 1.08)
Bachelor degree or graduate certificate	402	35.8	23.8 (20.3, 27.4)	1.20 (1.14, 1.26)
Postgraduate qualification	187	16.7	17.9 (13.4, 22.5)	0.97 (0.92, 1.03)
**Annual household income before tax (AUD; ref: <AUD 50,000)**	282	25.1	22.7 (18.5, 26.9)	
AUD 51,000–AUD 100,000	357	31.8	25.0 (21.5, 28.6)	1.07 (1.03, 1.11)
AUD 101,000–AUD 150,000	206	18.4	21.5 (17.9, 25.2)	1.07 (1.02, 1.12)
>AUD 150,000	214	19.1	18.3 (14.8, 21.9)	0.99 (0.93, 1.04)
**Financial situation (ref: Comfortable)**	353	31.5	19.9 (16.4, 23.3)	
Doing ok	480	42.8	21.8 (18.9, 24.8)	1.12 (1.08, 1.16)
Getting by	194	17.3	22.0 (18.4, 25.5)	1.12 (1.07, 1.17)
Difficult or very difficult	95	8.5	29.4 (23.2, 35.6)	1.43 (1.36, 1.50)
**Nature relatedness scale (ref: Tertile 1 (1.0–3.4))**	514	45.8	22.1 (19.5, 24.7)	
Tertile 2 (3.5–4.0)	331	29.5	20.2 (17.0, 23.5)	0.93 (0.90, 0.96)
Tertile 3 (4.1–5.0)	277	24.7	24.2 (19.8, 28.6)	0.98 (0.95, 1.01)
***Random Effects*** Unadjusted mean (95% CrI) ^†^			0.05 (0.03, 0.11)	
Adjusted mean (95% CrI)				0.05 (0.02, 0.10)
Percentage reduction in variance				14.6

IRR: Incidence Rate Ratio. OR: Odds Ratio. 95% CrI: 95% Credible Interval. Probabilistic weights applied to descriptive means, percentages and 95% CI (95% Confidence Intervals). ^†^ estimates derived from Variance Components Model. Models additionally adjusted for country of birth, household structure, household type, access to a private garden or balcony, and dog ownership.

**Table 3:** Associations with visits to preferred green or blue spaces during the COVID-19 pandemic.

	N	%	Total Hours in Green/Blue Space in the Last 7 Days		N Types of Green/Blue Spaces Visited in the Last 7 Days	
			Mean (95% CI)	IRR (95% CrI)	Mean (95% CI)	IRR (95% CrI)
Full sample	2697	100	3.2 (3.1, 3.3)		1.6 (1.5, 1.7)	
***Fixed Effects*** **Gender (ref: Female)**	1217	45.1	3.3 (3.1, 3.5)		1.7 (1.6, 1.8)	
Male	1483	54.9	3.1 (3.0, 3.3)	0.79 (0.76, 0.81)	1.5 (1.4, 1.6)	0.93 (0.87, 0.99)
**Age group (ref: 18–24)**	103	3.8	2.1 (1.7, 2.6)		1.2 (1.0, 1.5)	
25–34	356	13.2	3.0 (2.8, 3.3)	1.48 (1.30, 1.69)	1.5 (1.4, 1.7)	1.09 (0.89, 1.32)
35–44	448	16.6	3.1 (2.9, 3.4)	1.78 (1.56, 2.03)	1.5 (1.4, 1.7)	1.19 (0.98, 1.44)
45–54	458	17.0	3.0 (2.7, 3.2)	1.52 (1.33, 1.73)	1.7 (1.5, 1.9)	1.19 (0.97, 1.44)
55–64	549	20.3	3.5 (3.3, 3.8)	2.03 (1.78, 2.31)	1.6 (1.4, 1.7)	1.12 (0.92, 1.37)
65–74	541	20.0	4.1 (3.9, 4.4)	2.14 (1.86, 2.45)	1.7 (1.6, 1.9)	1.08 (0.87, 1.34)
75+	245	9.1	3.9 (3.5, 4.3)	1.96 (1.68, 2.25)	1.8 (1.6, 2.1)	1.09 (0.86, 1.37)
**Economic status (ref: Employed, not working from home)**	680	25.2	2.9 (2.6, 3.1)		1.4 (1.3, 1.5)	
Employed, working from home sometimes	285	10.6	3.2 (2.9, 3.6)	1.06 (1.00, 1.12)	1.6 (1.4, 1.9)	1.08 (0.97, 1.21)
Employed, working from home often	201	7.4	3.4 (3.0, 3.8)	1.01 (0.94, 1.08)	1.8 (1.6, 2.0)	1.27 (1.13, 1.43)
Employed, working from home always	380	14.1	3.3 (3.0, 3.7)	1.19 (1.13, 1.26)	1.8 (1.6, 2.0)	1.14 (1.03, 1.26)
Unemployed	188	7.0	2.6 (2.2, 3.1)	0.98 (0.90, 1.06)	1.3 (1.1, 1.6)	1.05 (0.91, 1.21)
Retired	787	29.2	4.1 (3.9, 4.3)	1.05 (0.99, 1.11)	1.8 (1.7, 2.0)	1.30 (1.15, 1.46)
Long-term sick or disabled	73	2.7	2.4 (1.5, 3.2)	0.79 (0.70, 0.90)	0.8 (0.6, 1.1)	0.75 (0.58, 0.95)
**Highest educational qualification (ref: <Year 12)**	345	12.8	3.2 (2.9, 3.5)		1.4 (1.3, 1.6)	
Year 12	332	12.3	2.7 (2.4, 3.0)	0.88 (0.82, 0.94)	1.4 (1.3, 1.6)	1.03 (0.91, 1.17)
Certificate or advanced diploma	744	27.6	3.3 (3.1, 3.5)	0.96 (0.91, 1.01)	1.6 (1.4, 1.7)	1.07 (0.96, 1.18)
Bachelor degree or graduate certificate	859	31.8	3.4 (3.1, 3.6)	1.01 (0.96, 1.06)	1.7 (1.6, 1.8)	1.21 (1.09, 1.33)
Postgraduate qualification	420	15.6	3.8 (3.5, 4.1)	0.99 (0.93, 1.06)	1.9 (1.8, 2.1)	1.32 (1.18, 1.48)
**Annual household income before tax (AUD; ref: <AUD 50,000)**	776	28.7	3.3 (3.1, 3.5)		1.5 (1.4, 1.6)	
AUD 51,000–AUD 100,000	842	31.2	3.1 (2.8, 3.3)	0.98 (0.94, 1.02)	1.5 (1.3, 1.6)	1.05 (0.96, 1.14)
AUD 101,000–AUD 150,000	468	17.3	3.1 (2.8, 3.4)	0.92 (0.86, 0.97)	1.7 (1.5, 1.9)	1.11 (0.99, 1.23)
>AUD 150,000	441	16.3	3.6 (3.3, 3.9)	1.05 (0.98, 1.11)	1.8 (1.6, 2.0)	1.15 (1.02, 1.29)
**Financial situation (ref: Comfortable)**	825	30.6	3.6 (3.4, 3.8)		1.8 (1.6, 1.9)	
Doing ok	1156	42.8	3.2 (3.0, 3.4)	0.89 (0.86, 0.93)	1.6 (1.5, 1.7)	0.96 (0.89, 1.03)
Getting by	491	18.2	3.0 (2.7, 3.2)	0.80 (0.76, 0.84)	1.5 (1.3, 1.6)	0.88 (0.80, 0.97)
Difficult or very difficult	228	8.4	2.8 (2.4, 3.1)	0.79 (0.74, 0.85)	1.4 (1.1, 1.6)	0.83 (0.72, 0.94)
**Nature relatedness scale (ref: Tertile 1 (1.0–3.4))**	884	32.7	2.6 (2.5, 2.8)		1.3 (1.2, 1.4)	
Tertile 2 (3.5–4.0)	1082	40.1	3.5 (3.2, 3.7)	1.42 (1.37, 1.48)	1.8 (1.6, 1.9)	1.32 (1.23, 1.42)
Tertile 3 (4.1–5.0)	734	27.2	4.0 (3.8, 4.2)	1.81 (1.74, 1.88)	1.9 (1.8, 2.0)	1.47 (1.37, 1.58)
***Random Effects*** Unadjusted mean (95% CrI) ^†^			0.12 (0.05, 0.26)		0.01 (<0.01, 0.02)	
Adjusted mean (95% CrI)				0.05 (0.02, 0.10)		0.01 (0.00, 0.02)
Percentage reduction in variance				61.4		-

OR: Odds Ratio. IRR: Incidence Rate Ratio. 95% CrI: 95% Credible Interval. Probabilistic weights applied to descriptive means, percentages and 95% CI (95% Confidence Intervals). ^†^ estimates derived from Variance Components Model. Models additionally adjusted for country of birth, household structure, household type, access to a private garden or balcony, and dog ownership.

**Table 4:** Associations with visits to green or blue spaces during the COVID-19 pandemic.

	N	%	Visited Preferred Green/Blue Space at Least Once a Week in Last 4 Weeks		Visit Green/Blue Spaces More Often Now than before COVID	
			% Yes (95% CI)	OR (95% CrI)	% Yes (95% CI)	OR (95% CrI)
Full sample	2697	100	52.0 (49.6, 54.4)		27.6 (25.5, 29.9)	
***Fixed Effects*** **Gender (ref: Female)**	1217	45.1	53.2 (49.7, 56.7)		26.9 (23.8, 30.3)	
Male	1483	54.9	50.8 (47.4, 54.2)	0.88 (0.74, 1.04)	28.3 (25.4, 31.5)	1.18 (0.97, 1.44)
**Age group (ref: 18–24)**	103	3.8	42.0 (32.0, 52.7)		37.3 (27.7, 48.0)	
25–34	356	13.2	47.7 (41.4, 53.9)	1.33 (0.78, 2.13)	37.2 (31.4, 43.5)	1.00 (0.59, 1.63)
35–44	448	16.6	46.5 (40.9, 52.3)	1.45 (0.84, 2.33)	34.4 (29.2, 40.0)	0.78 (0.45, 1.27)
45–54	458	17.0	51.5 (46.1, 56.9)	1.61 (0.93, 2.57)	21.7 (17.7, 26.4)	0.53 (0.30, 0.87)
55–64	549	20.3	56.4 (51.5, 61.3)	1.76 (1.02, 2.82)	16.3 (13.1, 20.2)	0.40 (0.22, 0.65)
65–74	541	20.0	65.4 (60.5, 70.1)	1.65 (0.89, 2.76)	19.8 (16.1, 24.2)	0.48 (0.24, 0.82)
75+	245	9.1	59.8 (52.2, 66.9)	1.32 (0.66, 2.34)	21.5 (15.9, 28.4)	0.46 (0.21, 0.84)
**Economic status (ref: Employed, not working from home)**	680	25.2	47.6 (43.0, 52.3)		24.4 (20.4, 28.7)	
Employed, working from home sometimes	285	10.6	45.6 (38.7, 52.8)	0.97 (0.71, 1.29)	27.6 (21.5, 34.6)	1.20 (0.84, 1.67)
Employed, working from home often	201	7.4	52.1 (43.4, 60.6)	1.24 (0.87, 1.72)	30.4 (23.2, 38.7)	1.52 (1.02, 2.16)
Employed, working from home always	380	14.1	55.6 (49.1, 61.9)	1.13 (0.85, 1.49)	47.8 (41.4, 54.3)	2.01 (1.46, 2.71)
Unemployed	188	7.0	53.1 (44.0, 61.9)	1.54 (1.06, 2.17)	28.7 (20.9, 38.1)	1.05 (0.67, 1.54)
Retired	787	29.2	64.4 (60.2, 68.4)	1.91 (1.35, 2.64)	19.4 (16.3, 22.9)	1.14 (0.74, 1.69)
Long-term sick or disabled	73	2.7	30.5 (19.0, 45.1)	0.60 (0.33, 0.99)	13.3 (7.1, 23.7)	0.86 (0.39, 1.58)
**Highest qualification (ref: <Year 12)**	345	12.8	53.9 (47.9, 59.8)		18.1 (14.0, 23.0)	
Year 12	332	12.3	46.2 (39.8, 52.6)	1.00 (0.70, 1.39)	28.4 (22.6, 35.0)	0.83 (0.54, 1.22)
Certificate or advanced diploma	744	27.6	51.7 (47.5, 55.9)	1.05 (0.78, 1.38)	23.3 (19.8, 27.1)	0.93 (0.65, 1.29)
Bachelor degree or graduate certificate	859	31.8	52.9 (48.1, 57.6)	1.38 (1.02, 1.82)	38.7 (34.2, 43.4)	1.26 (0.90, 1.75)
Postgraduate qualification	420	15.6	60.6 (54.4, 66.5)	1.64 (1.16, 2.26)	34.9 (29.2, 41.1)	1.12 (0.75, 1.61)
**Annual household income before tax (AUD; ref: <AUD 50,000)**	776	28.7	52.3 (47.8, 56.8)		23.9 (20.1, 28.1)	
AUD 51,000–AUD 100,000	842	31.2	49.4 (45.1, 53.8)	1.14 (0.89, 1.42)	22.9 (19.3, 26.9)	0.88 (0.66, 1.15)
AUD 101,000–AUD 150,000	468	17.3	50.7 (45.0, 56.5)	1.02 (0.75, 1.35)	32.4 (27.2, 38.0)	1.16 (0.81, 1.60)
>AUD 150,000	441	16.3	57.6 (51.6, 63.4)	1.18 (0.84, 1.61)	33.6 (28.2, 39.4)	1.05 (0.71, 1.50)
**Financial situation (ref: Comfortable)**	825	30.6	58.9 (54.4, 63.2)		27.8 (23.9, 32.0)	
Doing ok	1156	42.8	52.5 (48.7, 56.2)	0.83 (0.68, 1.01)	28.5 (25.2, 32.1)	1.08 (0.85, 1.35)
Getting by	491	18.2	44.0 (38.5, 49.5)	0.62 (0.47, 0.79)	28.4 (23.5, 34.0)	1.21 (0.88, 1.61)
Difficult or very difficult	228	8.4	45.3 (37.5, 53.3)	0.56 (0.39, 0.78)	21.4 (15.2, 29.4)	0.77 (0.49, 1.14)
**Nature relatedness scale (ref: Tertile 1 (1.0–3.4))**	884	32.7	42.6 (39.0, 46.3)		25.2 (22.0, 28.7)	
Tertile 2 (3.5–4.0)	1082	40.1	57.5 (52.9, 61.9)	1.79 (1.47, 2.16)	31.1 (27.0, 35.5)	1.38 (1.10, 1.71)
Tertile 3 (4.1–5.0)	734	27.2	62.5 (57.9, 66.9)	2.06 (1.67, 2.52)	28.0 (23.9, 32.4)	1.36 (1.06, 1.70)
***Random Effects*** Unadjusted mean (95% CrI) ^†^			0.06 (0.02, 0.16)		0.46 (0.16, 1.13)	
Adjusted mean (95% CrI)				0.07 (0.02, 0.19)		0.31 (0.10, 0.82)
Percentage reduction in variance				-		30.8

OR: Odds Ratio. IRR: Incidence Rate Ratio. 95% CrI: 95% Credible Interval. Probabilistic weights applied to descriptive means, percentages and 95% CI (95% Confidence Intervals). ^†^ estimates derived from Variance Components Model. Models additionally adjusted for country of birth, household structure, household type, access to a private garden or balcony, and dog ownership.

**Table 5:** Associations with social connection and solace in green/blue spaces during the COVID-19 pandemic.

	N	%	Green Spaces and/or Blue Spaces Have Helped Me to Stay Connected during the Pandemic		Green Spaces and/or Blue Spaces Have Brought Me Solace and Respite in These Challenging Times	
			% Yes (95% CI)	OR (95% CrI)	% Yes (95% CI)	OR (95% CrI)
Full sample N	2697	100	25.7 (23.7, 27.9)		53.7 (51.3, 56.1)	
***Fixed Effects*** **Gender (ref: Female)**	1217	45.1	23.0 (20.2, 26.1)		47.5 (44.0, 51.0)	
Male	1483	54.9	28.4 (25.5, 31.4)	1.47 (1.21, 1.76)	59.8 (56.4, 63.1)	1.74 (1.45, 2.06)
**Age group (ref: 18–24)**	103	3.8	15.0 (9.0, 23.8)		52.4 (41.8, 62.7)	
25–34	356	13.2	27.6 (22.3, 33.6)	1.84 (0.95, 3.43)	65.0 (58.8, 70.7)	1.32 (0.77, 2.12)
35–44	448	16.6	31.5 (26.3, 37.2)	2.20 (1.13, 4.12)	51.7 (45.9, 57.4)	0.78 (0.46, 1.24)
45–54	458	17.0	25.0 (20.6, 30.0)	1.86 (0.97, 3.48)	53.2 (47.7, 58.5)	0.82 (0.48, 1.30)
55–64	549	20.3	21.2 (17.6, 25.3)	1.76 (0.90, 3.30)	49.2 (44.3, 54.1)	0.79 (0.46, 1.25)
65–74	541	20.0	27.0 (22.7, 31.8)	2.25 (1.08, 4.40)	51.2 (46.0, 56.3)	0.73 (0.39, 1.21)
75+	245	9.1	30.5 (24.1, 37.7)	3.10 (1.37, 6.32)	44.8 (37.6, 52.3)	0.54 (0.27, 0.96)
**Economic status (ref: Employed, not working from home)**	680	25.2	24.4 (20.5, 28.7)		49.1 (44.4, 53.8)	
Employed, working from home sometimes	285	10.6	27.3 (21.2, 34.3)	1.19 (0.84, 1.64)	54.4 (47.2, 61.4)	1.36 (0.99, 1.82)
Employed, working from home often	201	7.4	26.9 (20.1, 34.9)	1.40 (0.95, 1.98)	56.8 (48.0, 65.2)	1.57 (1.08, 2.20)
Employed, working from home always	380	14.1	29.7 (24.2, 36.0)	1.19 (0.86, 1.59)	65.2 (58.8, 71.0)	1.54 (1.13, 2.05)
Unemployed	188	7.0	24.3 (17.4, 32.9)	1.34 (0.87, 1.98)	52.3 (43.3, 61.2)	1.28 (0.88, 1.82)
Retired	787	29.2	27.0 (23.5, 30.9)	1.22 (0.83, 1.73)	50.1 (45.9, 54.3)	1.47 (1.05, 2.02)
Long-term sick or disabled	73	2.7	12.9 (7.0, 22.4)	1.13 (0.54, 2.01)	46.5 (32.6, 60.9)	1.55 (0.86, 2.58)
**Highest qualification (ref: <Year 12)**	345	12.8	20.4 (16.2, 25.3)		41.9 (36.2, 47.9)	
Year 12	332	12.3	22.2 (17.5, 27.8)	1.35 (0.90, 1.96)	50.3 (43.9, 56.7)	1.16 (0.82, 1.62)
Certificate or advanced diploma	744	27.6	26.4 (22.8, 30.4)	1.33 (0.95, 1.82)	52.2 (48.0, 56.3)	1.33 (0.99, 1.75)
Bachelor degree or graduate certificate	859	31.8	29.8 (25.7, 34.2)	1.54 (1.10, 2.10)	63.5 (58.9, 67.8)	1.77 (1.32, 2.34)
Postgraduate qualification	420	15.6	29.2 (23.9, 35.1)	1.53 (1.04, 2.16)	63.6 (57.5, 69.3)	1.84 (1.31, 2.54)
**Annual household income before tax (AUD; ref: <AUD 50,000)**	776	28.7	24.9 (21.2, 28.9)		50.4 (45.9, 54.9)	
AUD 51,000–AUD 100,000	842	31.2	22.2 (18.9, 25.9)	0.97 (0.75, 1.25)	50.4 (46.0, 54.8)	1.06 (0.83, 1.33)
AUD 101,000–AUD 150,000	468	17.3	30.8 (25.7, 36.4)	1.11 (0.79, 1.51)	53.8 (48.0, 59.5)	1.13 (0.83, 1.51)
>AUD 150,000	441	16.3	29.3 (24.0, 35.1)	1.06 (0.73, 1.50)	64.2 (58.3, 69.6)	1.36 (0.95, 1.88)
**Financial situation (ref: Comfortable)**	825	30.6	27.9 (24.1, 32.1)		58.9 (54.5, 63.1)	
Doing ok	1156	42.8	26.9 (23.8, 30.4)	1.09 (0.87, 1.34)	52.6 (48.9, 56.4)	1.01 (0.81, 1.23)
Getting by	491	18.2	24.7 (20.3, 29.8)	1.00 (0.75, 1.32)	50.6 (44.9, 56.2)	0.92 (0.70, 1.19)
Difficult or very difficult	228	8.4	15.1 (10.4, 21.5)	0.59 (0.37, 0.86)	49.6 (41.6, 57.6)	0.73 (0.50, 1.02)
**Nature relatedness scale (ref: Tertile 1 (1.0–3.4))**	884	32.7	19.0 (16.3, 22.0)		38.1 (34.5, 41.7)	
Tertile 2 (3.5–4.0)	1082	40.1	27.0 (23.3, 31.0)	1.65 (1.32, 2.04)	63.1 (58.7, 67.3)	2.94 (2.40, 3.56)
Tertile 3 (4.1–5.0)	734	27.2	36.3 (31.9, 41.0)	2.27 (1.79, 2.81)	70.7 (66.5, 74.6)	3.73 (3.01, 4.59)
***Random Effects*** Unadjusted random effect mean (95% CrI) ^†^			0.06 (0.01, 0.17)		0.07 (0.02, 0.22)	
Adjusted random effect mean (95% CrI)				0.05 (0.01, 0.18)		0.07 (0.01, 0.23)
Percentage reduction in variance				1.4		4.6

OR: Odds Ratio. 95% CrI: 95% Credible Interval. Probabilistic weights applied to descriptive means, percentages and 95% CI (95% Confidence Intervals). ^†^ estimates derived from Variance Components Model. Models additionally adjusted for country of birth, household structure, household type, access to a private garden or balcony, and dog ownership.

**Table 6:** Associations with exercise in and discovery of new green/blue spaces during the COVID-19 pandemic.

	N	%	I Now Walk and/or Exercise in Green Spaces and/or Blue Spaces More Often than Before COVID		I Have Discovered and Visited Green/Blue Spaces That I Had Not Visited Before the Pandemic	
			% Yes (95% CI)	OR (95% CrI)	% Yes (95% CI)	OR (95% CrI)
Full sample N	2697	100	28.2 (26.0, 30.5)		27.1 (24.9, 29.5)	
***Fixed Effects*** **Gender (ref: Female)**	1217	45.1	27.1 (24.0, 30.4)		25.2 (22.1, 28.6)	
Male	1483	54.9	29.3 (26.2, 32.5)	1.19 (0.98, 1.44)	29.0 (25.9, 32.2)	1.24 (1.02, 1.50)
**Age group (ref: 18–24)**	103	3.8	38.6 (28.9, 49.3)		40.8 (30.9, 51.6)	
25–34	356	13.2	39.7 (33.7, 45.9)	1.02 (0.58, 1.65)	37.5 (31.6, 43.8)	0.79 (0.47, 1.27)
35–44	448	16.6	30.7 (25.7, 36.1)	0.69 (0.39, 1.13)	34.3 (29.1, 39.9)	0.62 (0.36, 0.99)
45–54	458	17.0	22.8 (18.7, 27.5)	0.56 (0.32, 0.93)	20.6 (16.6, 25.2)	0.36 (0.21, 0.58)
55–64	549	20.3	16.3 (13.0, 20.1)	0.39 (0.22, 0.65)	15.1 (12.0, 18.8)	0.29 (0.17, 0.47)
65–74	541	20.0	21.5 (17.6, 26.0)	0.54 (0.28, 0.95)	17.7 (14.1, 22.1)	0.38 (0.20, 0.66)
75+	245	9.1	25.0 (19.0, 32.2)	0.56 (0.26, 1.03)	17.4 (12.4, 23.7)	0.40 (0.19, 0.74)
**Economic status (ref: Employed, not working from home)**	680	25.2	25.2 (21.3, 29.7)		27.0 (22.9, 31.6)	
Employed, working from home sometimes	285	10.6	28.0 (21.8, 35.1)	1.11 (0.77, 1.52)	29.8 (23.5, 36.9)	1.26 (0.89, 1.72)
Employed, working from home often	201	7.4	33.9 (26.4, 42.4)	1.61 (1.10, 2.27)	29.4 (22.3, 37.8)	1.32 (0.89, 1.89)
Employed, working from home always	380	14.1	43.5 (37.2, 50.0)	1.66 (1.20, 2.22)	41.4 (35.2, 48.0)	1.43 (1.03, 1.92)
Unemployed	188	7.0	31.6 (23.7, 40.7)	1.19 (0.78, 1.74)	27.9 (19.9, 37.6)	0.80 (0.50, 1.19)
Retired	787	29.2	20.7 (17.5, 24.3)	1.07 (0.71, 1.55)	16.1 (13.2, 19.4)	0.84 (0.55, 1.26)
Long-term sick or disabled	73	2.7	14.1 (7.7, 24.3)	1.01 (0.48, 1.80)	15.4 (8.6, 26.0)	1.16 (0.55, 2.11)
**Highest qualification (ref: <Year 12)**	345	12.8	22.0 (17.3, 27.4)		16.3 (12.4, 21.2)	
Year 12	332	12.3	31.1 (25.3, 37.7)	0.84 (0.56, 1.23)	28.2 (22.5, 34.8)	1.01 (0.66, 1.50)
Certificate or advanced diploma	744	27.6	22.7 (19.3, 26.6)	0.77 (0.54, 1.08)	23.3 (19.7, 27.3)	1.01 (0.70, 1.42)
Bachelor degree or graduate certificate	859	31.8	36.8 (32.4, 41.4)	1.15 (0.82, 1.60)	36.1 (31.6, 40.8)	1.27 (0.89, 1.77)
Postgraduate qualification	420	15.6	35.6 (29.8, 41.8)	1.05 (0.71, 1.51)	37.7 (31.7, 44.1)	1.42 (0.96, 2.07)
**Annual household income before tax (AUD; ref: <AUD 50,000)**	776	28.7	26.1 (22.1, 30.4)		21.7 (18.0, 25.8)	
AUD 51,000–AUD 100,000	842	31.2	24.6 (21.0, 28.6)	1.01 (0.76, 1.30)	22.0 (18.6, 25.9)	1.09 (0.82, 1.43)
AUD 101,000–AUD 150,000	468	17.3	30.3 (25.3, 35.9)	1.13 (0.80, 1.57)	33.1 (27.9, 38.8)	1.33 (0.94, 1.86)
>AUD 150,000	441	16.3	34.8 (29.3, 40.7)	1.19 (0.81, 1.68)	36.4 (30.7, 42.5)	1.35 (0.91, 1.92)
**Financial situation (ref: Comfortable)**	825	30.6	29.3 (25.4, 33.5)		26.7 (22.9, 31.0)	
Doing ok	1156	42.8	28.7 (25.3, 32.3)	1.06 (0.85, 1.32)	28.5 (25.1, 32.1)	1.10 (0.88, 1.38)
Getting by	491	18.2	24.9 (20.3, 30.1)	1.00 (0.73, 1.32)	26.9 (22.0, 32.4)	1.14 (0.83, 1.53)
Difficult or very difficult	228	8.4	28.9 (21.8, 37.2)	0.89 (0.59, 1.30)	22.3 (15.9, 30.5)	0.85 (0.54, 1.26)
**Nature relatedness scale (ref: Tertile 1 (1.0–3.4))**	884	32.7	25.1 (22.0, 28.5)		23.3 (20.2, 26.8)	
Tertile 2 (3.5–4.0)	1082	40.1	32.2 (28.1, 36.7)	1.40 (1.12, 1.73)	28.2 (24.3, 32.5)	1.46 (1.16, 1.82)
Tertile 3 (4.1–5.0)	734	27.2	28.9 (24.7, 33.5)	1.33 (1.05, 1.67)	32.7 (28.2, 37.5)	1.65 (1.29, 2.07)
***Random Effects*** Unadjusted random effect mean (95% CrI) ^†^			0.40 (0.14, 0.98)		0.34 (0.10, 0.90)	
Adjusted random effect mean (95% CrI)				0.29 (0.10, 0.75)		0.21 (0.05, 0.63)
Percentage reduction in variance				30.0		61.1

OR: Odds Ratio. 95% CrI: 95% Credible Interval. Probabilistic weights applied to descriptive means, percentages and 95% CI (95% Confidence Intervals). ^†^ estimates derived from Variance Components Model. Models additionally adjusted for country of birth, household structure, household type, access to a private garden or balcony, and dog ownership.

## Data Availability

The data are not publically available.
